# MicroRNAs as Useful Tools to Estimate Time Since Death. A Systematic Review of Current Literature

**DOI:** 10.3390/diagnostics11010064

**Published:** 2021-01-03

**Authors:** Aniello Maiese, Andrea Scatena, Andrea Costantino, Marco Di Paolo, Raffaele La Russa, Emanuela Turillazzi, Paola Frati, Vittorio Fineschi

**Affiliations:** 1 Department of Surgical Pathology, Medical, Molecular and Critical Area, Institute of Legal Medicine, University of Pisa, 56126 Pisa (PI), Italy; aniello.maiese@unipi.it (A.M.); a.scatena2@studenti.unipi.it (A.S.); a.costantino8@studenti.unipi.it (A.C.); marco.dipaolo@unipi.it (M.D.P.); emanuela.turillazzi@unipi.it (E.T.); 2 IRCSS Neuromed Mediterranean Neurological Institute, Via Atinense 18, 86077 Pozzilli (IS), Italy; raffaele.larussa@uniroma1.it (R.L.R.); paola.frati@uniroma1.it (P.F.); 3 Department of Anatomical, Histological, Forensic and Orthopaedic Sciences, Sapienza University of Rome, Viale Regina Elena 336, 00161 Rome (RM), Italy

**Keywords:** post-mortem interval, miRNA, forensic medicine

## Abstract

Estimating the time of death remains the most challenging question in forensic medicine, because post-mortem interval (PMI) estimation can be a remarkably difficult goal to achieve. The aim of this review is to analyze the potential of microRNAs (miRNAs) to evaluate PMI. MiRNAs have been studied as hallmarks and biomarkers in several pathologies and have also showed interesting applications in forensic science, such as high sensible biomarkers in body fluid and tissue, for wound age determination and PMI evaluation due to their low molecular weight and tissue-specific expression. The present systematic review was carried out according to the Preferred Reporting Items for Systematic Review (PRISMA) standards. We performed an electronic search of PubMed, Science Direct Scopus, and Excerpta Medica Database (EMBASE) from the inception of these databases to 12 August 2020. The search terms were (“PMI miRNA” or “PMI micro RNA”) and (“miRNA” and “time of death”) in the title, abstract and keywords. Through analysis of scientific literature regarding forensic uses of miRNAs, has emerged that the intrinsic characteristics of such molecules, and their subsequent resistance to degradation, make them suitable as endogenous markers in order to determine PMI. However, further and larger studies with human samples and standardized protocols are still needed.

## 1. Introduction

Estimating the time of human death remains one of the most challenging question in forensic medicine [1]. Since ancient times, the determination of the time of death has struggled all professional figures involved around corpses because post-mortem interval (PMI) determination can be a remarkably difficult goal to achieve [2]. Forensic pathologists use different procedures to estimate the time of death: physical and physicochemical transformations, like body cooling, hypostasis and rigor mortis; autolysis postmortem metabolic and putrefactive processes [3,4]. In fact, quite soon after death, numerous processes begin at various times in the body [5]. Even if those changes are properly manifested, the accurate timing of these processes cannot be estimated because endogenous and exogenous factors can influence these modifications.

In the last years, scientists have described how many biological macromolecules in dead bodies, such as proteins, DNA and RNA, degrade by putrefaction as the PMI increases [6,7,8]. RNA is believed to be more predisposed to post-mortem and in vitro degradation than DNA due to the broad and ubiquitous presence of human and bacterial ribonucleases. [9,10,11]. MicroRNAs (miRNAs), are small non-coding RNAs of 18 to 24 nucleotides in length, are well preserved in eukaryotic organisms. MiRNAs’ role is to regulate the expression of many genes in several biological processes during post-transcriptional phase. MiRNAs bind complementary sequences of messenger RNA (mRNA) targets and silence them via degradation through mRNA cleavage or by inhibiting protein synthesis. In recent years, there was an outbreak of the use of micro-RNAs in clinical and forensic medicine [10].

The interest around miRNAs started when their functional ability was discovered. In 1993 Lee at al. discovered that miRNA lin-4 can regulate protein production lin-4 [12]. Let-7 was the second miRNA discovered [13] and miRNAs were consequently recognized as a new category of riboregulators [14,15]. The real revolution in this new field arose when researchers found out that miRNAs play crucial roles in a wide range of biological and pathological pathways and that they are tissue-specific [16,17,18,19,20].

Since that moment, researchers mainly focused on the role of miRNAs in clinical pathology due to their high specificity for certain tissues. MiRNAs have been studied as hallmarks and biomarkers in several pathologies, such as diabetes [21], cancer [22], cardiovascular disease [23], muscular disorders [24] and spinal cord injury [25]. Lately Hanson et al. had the first suggestion of the potential implication of miRNAs in the forensic field, using them to identify body fluids [26]. As a matter of fact, miRNAs showed interesting applications in this field, such as high sensible biomarkers in body fluid and tissue identification, for wound age determination [27].

Recently, Odriozola et al. analyzed miRNAs in vitreous humor and the data obtained supported the suggestion to use miRNAs for PMI assessment [28] due to their low molecular weight and tissue-specific expression. In fact, most miRNAs are sited inside the cell, even though some of them have also been discovered in various biological fluids like plasma, tears, cerebrospinal fluid and saliva [10]. The principal difference between miRNAs and other macromolecules, such as DNA, RNA and proteins, is the high stability even at extreme condition of temperatures, pH and chemical treatments [10].

The availability of innovative technologies has allowed high multiplexing with great sensitivity [15]. The analysis of miRNAs can be achieved with microarray or Next Generation Sequencing (NGS) techniques that permits hundreds of miRNAs to be identified even in very low copy number simultaneously in a single experiment. After their identification, researcher can perform the assay by quantitative Real-Time PCR/qRT-PCR) [10]. The aim of this review is to analyze the potential of microRNAs to evaluate PMI.

## 2. Materials and Methods

### 2.1. Eligibility Criteria

The present systematic review was carried out according to the Preferred Reporting Items for Systematic Review (PRISMA) standards [29]. We used an evidence-based model for framing a PICO question model (PICO: population, intervention, control, and outcomes).

### 2.2. Search Criteria and Critical Appraisal

A systematic literature search and a critical appraisal of the collected studies were conducted. An electronic search of PubMed, Science Direct Scopus, and Excerpta Medica Database (EMBASE) from the inception of these databases to 12 November 2020 was performed.

The search terms were (“PMI miRNA” or “PMI micro RNA”) and (“mirna” and “time of death”) in the title, abstract and keywords. Bibliographies of all identified documents were reviewed and compared for further relevant literature. Methodological evaluation of each study was conducted according to the PRISMA standards, including assessment of bias. Data collection involved study selection and data extraction. Three researchers (M.D.P., E.T., P.F.) independently reviewed those documents whose title or abstract appeared to be relevant and selected those who analysed the miRNAs PMI. Disagreements concerning eligibility between the researchers were resolved by consensus process. No unpublished or grey literature was searched. One investigator (A.M.) performed data extraction and another investigator (V.F.) verified it. Only papers or abstract in English were included in the search.

### 2.3. Search Results and Included Studies

An appraisal based on titles and abstracts, as well as a hand search of reference lists were carried out. The reference lists of all located articles were reviewed to detect still unidentified literature. This search identified 87 articles, that were then screened based on abstracts to identify their relevance in respect to the following:

- use of miRNAs in PMI determination;
- postmortem findings;
- study design.

**Figure 1:**
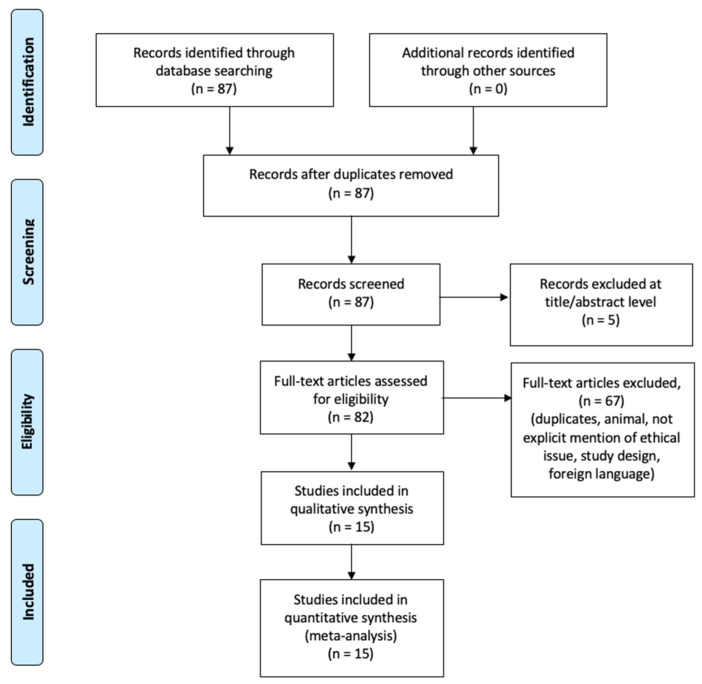
Preferred Reporting Items for Systematic Review (PRISMA) flow chart—search strategy.

The methodology of our search strategy is presented in Figure 1.

### 2.4. Risk of Bias

This systematic review has a number of strengths that include the number and breadth of the studies collected, which span the globe; the hand search and scan of reference lists for the identification of all relevant studies; and a flowchart that describe in detail the study selection process. It must be noted that this review includes studies that were published in a time frame of 15 years; thus, despite our efforts to fairly evaluate the existing literature, study results should be interpreted taking into account that the accuracy of the clinical procedures, where reported, has changed over the years.

## 3. Results

Study designs comprised retrospective and prospective studies original articles and reviews. The reference lists of all located articles were reviewed to detect still unidentified literature. A total of 15 studies fulfilled the inclusion criteria (Table 1).

## 4. Discussion

### 4.1. Current Methods of PMI Estimation

The post-mortem interval (PMI describes the estimated time between the discovery of a cadaver and the time of death. Estimation of this interval is a focal point of investigation in forensic field due to its civil and criminal implications. Despite of massive studies on this theme have been conducted for more than a century, the estimation of the PMI still remains challenging, even for expert pathologists [42]. Presently the estimation of the PMI is founded on the assessment of physical (algor mortis, livor mortis), physicochemical (rigor mortis), biochemical (electrolyte concentration, enzyme activity), microbiological (decomposition), entomological and botanical parameters [3,43]. All the procedures used to obtain the time of death are not totally accurate and they only offer a simple approximation because there are numerous variables which can influence the estimation of the PMI, such as environment temperature, location of the body, body structure or cause of death. These methods have limited practical relevance even when are used to the very early post-mortem period [42,44].

Shortly after death, several alterations to the corpse occur at different times [5]. In spite of massive studies about these transformations, the precise time of death remains hard to evaluate because endogenous and exogenous factors can heavily influence it, like temperature, humidity, age of the deceased, use of medications, and health conditions [1,2,5,45,46,47,48]. Unfortunately, this scenario becomes more and more problematic as time goes by and PMI increases. All the available literature data about PMI can be summarized in two categories: the early post-mortem changes and late postmortem changes periods [49]. If the dead body is discovered within 24 h after the fatal event, this situation is called early PMI (EPMI) and it can be determined evaluating physical and biochemical changes occurring short after death such as body temperature, post-mortem rigidity and lividity, degree of putrefaction, corneal cloudiness and characterization of gastric contents [50,51]. Instead, after the 24-h time frame, the late post-mortem period can be estimated with different strategies such as the forensic entomology. In fact, by knowing the lifecycle of insects that colonize the corpse and the sequence of colonizing insects, the entomologists can obtain longer PMIs. However, these evidences maintain a certain degree of inaccuracy, thus, attention must be paid in interpreting such findings, and, case-by-case evaluation is mandatory, because these environmental changes are context-dependent [37,52,53].

Scientific attempts should be made to substitute the traditional techniques of PMI estimation with parametric calculation, providing mean values and confidence limits, and including reliability and accuracy of calculation. For some procedures, Henssge nomograms provide the first step in this direction, but other careful and accurate methods for defining the PMI are surely desirable [18].

New approaches such as flow cytometry, capillary zone electrophoresis, magnetic resonance spectroscopy and immunohistochemistry have been proposed to evaluate post-mortem changes, trying to extrapolate the PMI [54]. These methods alone do not make on their own death time estimation more specific, but the combination of different approaches has been recommended in order to decrease the intrinsic error rate of each method [44,54].

A precise assessment of the PMI requires the evaluation of parameters that repeatedly change in time after death. This definition seems to fit well in post-mortem degradation of nucleic acids [55]. In fact, with the improvements of molecular biology, the analysis of time-dependent degradation of nucleic acids (both DNA and RNA) has become a focal point in clinical medicine as well as in forensic science [55,56].

### 4.2. Postmortem Changes in Biomolecules

In the last decade, researchers have explored a great number of experimental methodologies to precisely determine the time of death. The development of new scientific fields like molecular biology, has allowed the forensic pathologists to meticulously evaluate degradation rate of biological markers (e.g., proteins, DNA, and RNA) in order to obtain a more accurate estimation of PMI. Indeed, numerous researchers focused their work on the quantification of the degradation of macromolecules, such as DNA, RNA and proteins, as a possible indicator of PMI [56,57,58]. Due to its nature, RNA seems more accurate than DNA and proteins: its degeneration and the loss of specific RNA transcripts appear to be very susceptible in terms of rapidity and temporal correlation after the death of the organism [38].

### 4.3. RNA

After death, RNA is degraded by human ribonucleases, bacteria or environmental contamination. Therefore, its degradation depends not only on time, but also on other main factors, as the cause of death and environmental conditions [48,58].

Over the years, a large number of RNA species have been examined for the measurement of PMI, including messenger RNA (mRNA), ribosomal RNA (rRNA), and microRNA (miRNA). Currently, a wide range of tests is available, such as the real-time quantitative polymerase chain reaction (qRT-PCR), which is now considered the method of choice due to its high sensitivity [59,60]. However, this high quantitative accuracy may lead to misinterpretation of the data. In fact, sample processing can cause tiny changes in mRNAs levels that could be misinterpreted as variations in gene expression activity. For these reasons, the normalization of correct data is essential.

The best way for data standardization is the evaluation of endogenous reference genes, especially mRNA markers (including glyceraldehyde-3-phosphate dehydrogenase (GAPDH), β-actin, RPS-29, and IL-1β), nowadays considered suitable endogenous control markers in biochemical studies. Even if these molecular markers are routinely used, they degrade over time, losing some of their efficacy as indicators of PMI, particularly in uncertain environmental circumstances (i.e., high temperatures) [35,47,49,50,61].

Today we know that the degradation of RNA is influenced by several factors such as human ribonucleases or bacteria. This feature can be useful for determining PMI. If we can quantify RNA degradation in postmortem samples, we will indeed have a potential marker for PMI.

Real-time quantitative polymerase chain reaction (RT-qPCR) has been extensively used in PMI evaluations. Evidence shows that longer time since death is normally related to decreasing RNA transcription levels [58,62]. Researchers have already presented the quantification of degraded RNA as a potential marker in PMI determination [7,10,58]. However, postmortem RNA degradation in human cells depends not only on time, but also on other factors, such as environmental conditions [63]. Sampaio-Silva and colleagues developed a mathematical model with predictive value for estimation of the postmortem interval with 95% confidence interval of 651 min using RNA from murine’s visceral and muscle tissues. Researchers discovered four quadriceps muscle genes (Actb, Gapdh, Ppia and Srp72) that were found to significantly correlate with PMI using a quantitative evaluation of standardized transcript levels on the former tissues [58]. Bauer et colleagues tried to determine the time of death through the quantitative evaluation of mRNA degradation by multiplex-qPCR in combination with laser-induced fluorescence capillary electrophoresis. The data obtained indicated a substantial correlation between RNA degradation and post-mortem interval in stored refrigerated human blood and brain samples for up to 5 days [11]. On the contrary, other Authors have described a lack of significant correlation between mRNA degradation and PMI in human brain tissue [64,65].

The tests done so far, evaluating quantitatively the degradation of RNA as a post- mortem interval indicator, have only focused on limited mRNA transcripts, consequently achieving large confidence intervals [11,62]. Young et al. utilized RNA from tooth pulp to provide reasonably accurate evaluations of the time since death. Scientists demonstrated that β-actb RNA can be applied to estimate longer PMIs, up to 84 days [66].

Generally, researchers suppose that RNA degrades quickly after death, making it useless as a marker for the estimation of postmortem interval. Nevertheless, scientists have proved that RNA degradation in tissues is regulated by specific laws, and that RNA is quite stable after death [11,34,63].

The efforts to obtain an association between RNA degradation and PMI have not been totally successful, because appropriate and stable reference genes were absent for degraded tissues. Endogenous reference genes are crucial for internal control in data analysis due to RT-qPCR reliability. Housekeeping genes, rRNAs and small nuclear RNAs (snRNAs) have been regularly used as reference controls in fresh tissues. However, researchers have demonstrated that many of them revealed unpredictability in their expression levels regarding both the stability among various tissues, and different PMIs [46,67].

### 4.4. Current Literature about miRNAs in PMI

In recent times multiple studies have highlighted new potential miRNAs in PMI determination:

Li et al. observed variations of miR-1 and miR-2, in rat heart samples, characterized by different PMIs, stored at 25 °C. The results showed that the above-mentioned miRNAs are stable up to 120 h after death, before their levels slowly begin to decline. The same study also correlates miRNAs level with 18s rRNA in order to estimate PMI. [32,68]

Pan et al. correlated PMI and miR-203 and other RNA markers; they stored rat samples divided into three storage temperature groups: 4, 15 and 35 °C. This study concluded that miR-203 could be taken as an internal reference because of its good stability [33,68].

Lv et al. used rat spleen samples, creating two groups, one stored at 25 °C for 144 h, the other at 4 °C for 312 h. The researchers observed that miRNAs are suitable as endogenous controls in order to determine PMI; moreover the 25 °C group was more reliable due to the shortened time interval (the average error rate was lower than 10% in the 25 °C group versus an error rate lower than 20% in the 4 °C group). In this study it was also concluded that miR-125b was more stable than miR-143 for this purpose [34,68].

Ma et al. sacrificed 270 rats, collected their brain tissue and randomly divided the samples into a control group (PMI = 0 h) and four groups stored in a controlled-environment chamber at 4, 15, 25, and 35 °C, respectively. They then sampled brain tissue at different time points for up to 144 h PMI. Further analysis showed that miR-125b and miR-9 were, due to their high stability, effective endogenous control markers because they were not affected by PMI up to 144 h. Ma et al. used also β-actin as a factor correlated with PMI; in order to build a mathematical model that could estimate the time since death of rats at different temperatures correlating the concentration of the two molecules [35,68].

Lü et al. analyzed 222 rats’ brains, randomly divided into a control group and four experimental groups (5, 15, 25, and 35 °C). Samples were collected at nine time points between 1 and 24 h post-mortem and correlated with delta-Ct values, degradation of biomarkers (β-actin, GAPDH, RPS29, 18S rRNA, 5S rRNA, U6 snRNA, miRNA-9, and miRNA-125b) and endogenous control molecules (5S rRNA, miR-9 and miR-125b) [36,68].

Lv et al. evaluated at 12 time points (0–144 h), rat lung and muscle tissues. In this study miRNAs were used as reference biomarkers; in muscle samples miR-1 and miR-206 were used, whereas in lung samples miR-195 and miR-200c were used [37,68].

Lü et al. evaluated brain samples from 12 cadavers with known PMI from 4.3 to 22.5 h. Researchers concluded that miRNA-9 and miRNA-125b are suitable as internal reference markers of human brain tissue, due to their stable expression in early PMI. The authors also used the expression level of β-actin, that correlates well with PMI, which can be used as an additional index for early PMI estimation [69].

Lv et al., in 2017, took human heart, liver and brain samples from 13 dead human bodies with known PMI; moreover, the authors also sampled murine heart and liver tissues in order to verify the results. Among other molecules the researchers analyzed miR-1; miR-9; miR-133a; miR-133a; miR-1, miR-133a were shown to be an optimal reference biomarker due to the miRNA’s stability over five or more days; on the other hand miR-122 that is a liver-specific biomarker, began to degrade under higher temperatures. The authors concluded, using delta Ct with β-actin, that the error rate in six of 13 cases was lower than five hours for real PMI [38,68].

Tu et al. used 15 sacrificed healthy adult male mice, and heart, liver, and skeletal muscle tissue samples stored at 25 °C; the mice were then divided into five groups with different PMI values: 0, 1.5, 3.5, 5.5, and 7.5 d. The authors built a mathematical model for PMI estimation, using miR-122 as a reference biomarker for heart and liver and miR-133a for heart and skeletal muscle [31,68].

Wang et al. used cardiac muscle, liver, brain and skeletal muscle samples from 33 adult male mice, randomly grouped into 10 PMI time points within 48 h, with four mice in each group; all stored at −80 °C. The authors analyzed five miRNAs: miR-122, miR-133a, miR-150, miR-195, miR-206. MiR-133a and miR-206 were stable during the first 24 h, but after 24 h they began to decrease. MiR-195 has an anomalous behavior due to its increase even 24 h after death; this is likely because it’s still produced after death [30].

Tu et al. sacrificed 45 mice randomly divided into one control and eight experimental groups, each group with five mice, and used heart, liver and skeletal muscle samples stored at 25 °C. Tu et al. affirmed that miR-122 and miR-133a were more stable in degraded tissues (especially in heart); differently, in other tissues the behavior was discordant: miR-133a was more stable in skeletal muscle, and downregulated in liver, miR-122 was downregulated in skeletal muscle and stable in liver. MiR-122 was considered to be highly expressed specifically in liver; miR-133a, characterized in mice, corresponds to three genes in the human genome: miR-133a-1, miR-133a-2, miR-133b, located on chromosomes 18, 20 and 6, respectively. In conclusion, the authors affirmed that, for some tissues which were rich in hydrolytic enzymes, circRNAs might be a better marker choice [39].

Kuai et al. used male mice randomly divided into a control group (PMI = 0 h), and experimental groups (PMI = 1, 3, 6, 12, 15, 18, 21, 24, 36, 48, 72, 96, 120, 144, 168 h) (*n* = 6), killed and stored at 25 °C. Heart tissue was chosen because it was less affected by environmental conditions. In this study, miR-1 was explored as a stable endogenous marker to be used as an internal standard [40].

Odriozola et al. hypothesized that miRNAs could be used as a “biological black box”, i.e., as a tool to identify whether death occurred during the day or night. In order to confirm this theory authors used human vitreous humor samples in search of various miRNAs: miR-34c, miR-541, miR-888, miR-484, miR-142-5p, and miR-222. Among all, miR-222 showed to be the best single candidate as an internal reference biomarker. Other miRNAs (miR-541 and miR-142-p) showed differences between day and night, in particular they’re up-regulated in individuals who died at nighttime, and otherwise, were down-regulated in individuals who died during the day [9].

Sharma et al. employed five mice, collecting brain, heart, lungs, kidneys, pancreas, spleen and liver samples and storing them at 25 °C. The authors focused their attention on AATF mRNA and miRNA-2909; in conclusion was possible to deduce that miR-2909 was stable up to 48 h post-death if the mouse was sacrificed at 8 PM and 12 h post death if the mouse was sacrificed at noon. The authors also concluded that miR-2909 is much more stable to enzymatic degradation then AATF mRNA [41,70].

## 5. Conclusions

PMI has absolute medical-legal and investigative relevance, given the importance assigned to determining the time of death Despite all the scientific progress to date, the identification of the PMI is still a challenge for the forensic pathologist. There is, in fact, currently no way to give absolute and certain indications about the actual period of time elapsed since death, although one can try to restrict the thanatochronological interval as much as possible within scientifically acceptable and concrete investigative utility limits [71].

Through analysis of scientific literature regarding forensic uses of miRNAs, it emerged that the intrinsic characteristics of such molecules, and their subsequent resistance to degradation, make them suitable as housekeeping gene controls, so a suitable usage might be to create a mathematical model by comparing molecules with known degradation rates and miRNA concentrations, in specific tissues, obtaining a retrospective model.

Last but not least, miRNA research is not very expensive [72]; all the above mentioned features make miRNAs ideal candidates for daily practice in forensic laboratories, where it is the norm to work with degraded samples.

In fact, comparison between the degradation rate of endogenous molecules (such as DNA, RNA and proteins) and the concentration levels of more stable molecules (such as miRNAs) can be suggestive of a certain time lapse since death. As widely known, early PMI can be reliably assessed via numerous markers. As it is known, immediately after death, biological and environmental factors begin to fragment and degrade DNA molecules. The first studies about the application of degraded DNA, and DNA incorporation as biochemical or immunohistochemical methods of determining the PMI were published more than 30 years ago [73,74]. Furthermore, most of the data can only be applied effectively for short PMI estimations. Consequently, DNA degradation is considered of limited value in forensic investigations, when used to determine the PMI, as demonstrated in a recent review [70]. Similarly, due to technological advances, researchers are able to use many different methods to study the correlation between protein degradation rate and PMI [75,76,77,78,79]. Despite the fact the degradation rate of specific proteins has a considerable correlation with PMI, the protein degradation process is also biased by environmental temperature and putrefying bacteria, which hinder the application of this method in forensic practice.

The lack of consistent indicators for determining late PMI (>24 h), increases the potential of miRNAs as useful tools in this scenario. It should also be noted that Odriozola et al. have been able to use the relative concentrations of miR-541 and miR-142-p as a criterion to differentiate between daytime or nighttime deaths [28].

Currently, most studies, as shown in this review, deal with animal models, in particular rats and only few exceptions used human dead bodies. For this reason, it’s desirable that further and larger standardized studies with human samples be conducted. Due to this lack of human body-based studies, it’s also impossible to select a specific miRNA or a specific tissue to analyze in order establish PMI.

For all the above-mentioned reasons, miRNAs could become in the next future reliable forensic biomarkers for the diagnosis of PMI. Specifically, some miRNAs, which were discussed earlier in the present review, could become part of routine forensic practice integrating the current investigations.

## Figures and Tables

**Table 1:** Summary of the literature, including extracted data.

miRNA	Epicrisis of PMI	Tissues and Organs	Sample Source	Year	Authors [Reference]
miR-206	downregulated after 24 h	Liver	mice	2013	Wang, H.; et al. [30]
miR-150	downregulated after 24 h	Liver	mice	2013	Wang, H.; et al. [30]
miR-133	downregulated after 24 h	Liver	mice	2013	Wang, H.; et al. [30]
miR-122	downregulated after 24 h	Liver	mice	2013	Wang, H.; et al. [30]
miR-195	upregulated for first 24 h, then downregulated	Liver	mice	2013	Wang, H.; et al. [30]
miR-122	stable in heart tissue and liver; downregulated in skeletal muscle	heart, liver and skeletal muscle	mice	2018	Tu, C.; et al. [31]
miR-133a	stable in heart tissue; downregulated in skeletal muscle and liver	heart, liver and skeletal muscle	mice	2018	Tu, C.; et al. [31]
miR-1	stable for fist 120 h then downregulated	Heart	rat	2010	Li, W.C.; et al. [32]
miR-2	stable for fist 120 h then downregulated	Heart	rat	2010	Li, W.C.; et al. [32]
miR-203	miR-203 levels in this study were taken as internal reference because of his good stability.	Skin	rat	2014	Pan, H.; et al. [33]
miR-125b	the Ct values of miRs fluctuated slightly within 36 h and then increased slowly at 25 °C, with the same trend observed within 144 h at 4 °C. microRNAs are less susceptible to degradation induced by PMI and environmental conditions because of its short length of 21–25 bp.	Spleen	rat	2014	Lv, Y.H.; et al. [34]
miR-143	the Ct values of miRs fluctuated slightly within 36 h and then increased slowly at 25 °C, with the same trend observed within 144 h at 4 °C. microRNAs are less susceptible to degradation induced by PMI and environmental conditions because of its short length of 21–25 bp.	Spleen	rat	2014	Lv, Y.H.; et al. [34]
miR-9	stable up to 144 h post mortem	Brain	rat	2015	Ma, J.; et al. [35]
miR-125b	stable up to 144 h post mortem	Brain	rat	2015	Ma, J.; et al. [35]
miR-9	stable up to 24 h post mortem	Brain	rat	2016	Lü, Y.H.; et al. [36]
miR-125b	stable up to 24 h post mortem	Brain	rat	2016	Lü, Y.H.; et al. [36]
miR-195	chosen as control markers for muscle tissues because stable up to 144 h, then downregulated	lung; skeletal muscle	rat	2016	Lv, Y.H.; et al. [37]
miR-200c	chosen as control markers for muscle tissues because stable up to 144 h, then downregulated	lung; skeletal muscle	rat	2016	Lv, Y.H.; et al. [37]
miR-201	chosen as control markers for muscle tissues because stable up to 144 h, then downregulated	lung; skeletal muscle	rat	2016	Lv, Y.H.; et al. [37]
miR-206	chosen as control markers for muscle tissues because stable up to 144 h, then downregulated	lung; skeletal muscle	rat	2016	Lv, Y.H.; et al. [37]
miR-9	stable enough in order to be reference markers up to 22.5 h	Brain	dead body	2016	Lü, Y.H.; et al. [34]
miR-125b	stable enough in order to be reference markers up to 22.5 h	Brain	dead body	2016	Lü, Y.H.; et al. [34]
miR-1	myocardium-specific fairly stable over 5 or more days, even at 35 °C	myocardium, liver, brain	dead body	2017	Lv, Y.H.; et al. [38]
miR-133a	myocardium-specific fairly stable over 5 or more days, even at 35 °C	myocardium, liver, brain	dead body	2017	Lv, Y.H.; et al. [38]
miR-122	degrading after 4 days of PMI particularly at high temperatures	myocardium, liver, brain	dead body	2017	Lv, Y.H.; et al. [38]
miR-9	are suitable as internal reference markers of human brain tissue	myocardium, liver, brain	dead body	2017	Lv, Y.H.; et al. [38]
miR-125b	are suitable as internal reference markers of human brain tissue	myocardium, liver, brain	dead body	2017	Lv, Y.H.; et al. [38]
miR-122	stable up to 8 days choosen as reference control up to 7.5 d of PMI	heart, liver, skeletal muscle	rat	2019	Tu, C.; et al. [39]
miR-133a	stable up to 8 days choosen as reference control up to 7.5 d of PMI	heart, liver, skeletal muscle	rat	2019	Tu, C.; et al. [39]
miR-34c	CT downregulated with increase of PMI	vitreous humor	human	2013	Odriozola, A.; et al. [28]
miR-222	best reference gene, stable at least for 24 h	vitreous humor	human	2013	Odriozola, A.; et al. [28]
miR-888	almost stable up to 24 h post mortem, stable at least for 24 h	vitreous humor	human	2013	Odriozola, A.; et al. [28]
miR-484	CT downregulated with increase of PMI, stable at least for 24 h	vitreous humor	human	2013	Odriozola, A.; et al. [28]
miR-142-5p	upregulated in night time deaths, stable at least for 24 h	vitreous humor	human	2013	Odriozola, A.; et al. [28]
miR-541	upregulated in night time deaths, stable at least for 24 h	vitreous humor	human	2013	Odriozola, A.; et al. [28]
miR-miR-1	upregulated until 96 h	heart	rats	2014	Kuai, J.-X.; et al. [40]
miR-1	-	muscle	rat	2016	Lv, Y.H.; et al. [34]
miR-206	-	muscle	rat	2016	Lv, Y.H.; et al. [34]
miR-195	-	lung	rat	2016	Lv, Y.H.; et al. [34]
miR-2909	stable up to 48 h post death if the mouse was sacrificed at 8 PM and 12 h post death if the mouse was sacrificed at noon. They also concluded that miR-2909 is much more stable to enzymatic degradation then AATF mRNA	brain, heart, lungs, kidneys, pancreas, spleen and liver	mice	2014	Sharma, S.; et al. [41]

Abbreviations: PMI—Post Mortem Interval; CT—Cycle Threshold; AATF—Apoptosis Antagonizing Transcription Factor.

## Data Availability

No new data were created or analyzed in this study. Data sharing is not applicable to this article.

## Abbreviations

PRISMA	Preferred Reporting Items for Systematic Reviews and Meta-Analyses
PICO	Participants: Intervention, Control, and Outcomes
EMBASE	Excerpta Medica Database
PCR	Polymerase chain reaction
qRT-PCR	Quantitative real-time polymerase chain reaction
mRNA	Messenger RNA
rRNA	Ribosomal RNA
miRNA	MicroRNA

## References

[1] Buchan M.J., Anderson G.S. (2001). Time since death: A review of the current status of methods used in the later postmortem interval. J. Can. Soc. Forensic Sci..

[2] Sachs J.S. (2002). Corpse: Nature, Forensics, and the Struggle to Pinpoint Time of Death.

[3] Madea B. (1994). Importance of supravitality in forensic medicine. Forensic Sci. Int..

[4] Payne-James J., Smock W., Busuttil A. (2002). Forensic Medicine: Clinical and Pathological Aspects.

[5] Vass A.A., Barshick S.A., Sega G., Caton J., Skeen J.T., Love J.C., Sinstelien J.A. (2002). Decomposition chemistry of human remains: A new methodology for determining the postmortem interval. J. Forensic Sci..

[6] Kang S., Kassam N., Gauthier M.L., O’Day D.H. (2003). Postmortem changes in calmodulin binding proteins in muscle and lung. Forensic Sci. Int..

[7] Inoue H., Kimura A., Tuji T. (2002). Degradation profile of mRNA in a dead rat body: Basic semi-quantification study. Forensic Sci. Int..

[8] Larkin B., Iaschi S., Dadour I., Tay G.K. (2010). Using accumulated degree-days to estimate postmortem interval from the DNA yield of porcine skeletal muscle. Forensic Sci. Med. Pathol..

[9] Birdsill A.C., Walker D.G., Lue L.F., Sue L.I., Beach T.G. (2011). Postmortem interval effect on RNA and gene expression in human brain tissue. Cell Tissue Bank..

[10] Partemi S., Berne P.M., Batlle M., Berruezo A., Mont L., Riuró H., Ortiz J.T., Roig E., Pascali V.L., Brugada R. (2010). Analysis of mRNA from human heart tissue and putative applications in forensic molecular pathology. Forensic Sci. Int..

[11] Bauer M., Gramlich I., Polzin S., Patzelt D. (2003). Quantification of mRNA degradation as possible indicator of postmortem interval--a pilot study. Leg. Med..

[12] Lee R.C., Feinbaum R.L., Ambros V. (1993). The C. elegans heterochronic gene lin-4 encodes small RNAs with antisense complementarity to lin-14. Cell.

[13] Pasquinelli A.E., Reinhart B.J., Slack F., Martindale M.Q., Kuroda M.I., Maller B., Hayward D.C., Ball E.E., Degnan B., Müller P. (2000). Conservation of the sequence and temporal expression of let-7 heterochronic regulatory RNA. Nature.

[14] Lagos-Quintana M., Rauhut R., Lendeckel W., Tuschl T. (2001). Identification of novel genes coding for small expressed RNAs. Science.

[15] Lau N.C., Lim L.P., Weinstein E.G., Bartel D.P. (2001). An abundant class of tiny RNAs with probable regulatory roles in C. elegans. Science.

[16] Calin G.A., Dumitru C.D., Shimizu M., Bichi R., Zupo S., Noch E., Aldler H., Rattan S., Keating M., Rai K. (2002). Frequent deletions and down-regulation of micro- RNA genes miR15 and miR16 at 13q14 in chronic lymphocytic leukemia. Proc. Natl. Acad. Sci. USA.

[17] Ambros V. (2004). The functions of animal microRNAs. Nature.

[18] Bartel D.P. (2004). MicroRNAs: Genomics, biogenesis, mechanism, and function. Cell.

[19] Lu J., Getz G., Miska E.A., Alvarez-Saavedra E., Lamb J., Peck D., Sweet-Cordero A., Ebert B.L., Mak R.H., Ferrando A.A. (2005). MicroRNA expression profiles classify human cancers. Nature.

[20] Wegman D.W., Cherney L.T., Yousef G.M., Krylov S.N. (2013). Universal drag tag for direct quantitative analysis of multiple microRNAs. Anal. Chem..

[21] Lees T., Nassif N., Simpson A., Shad-Kaneez F., Martiniello-Wilks R., Lin Y., Jones A., Qu X., Lal S. (2017). Recent advances in molecular biomarkers for diabetes mellitus: A systematic review. Biomarkers.

[22] Croce C.M., Calin G.A. (2005). miRNAs, cancer, and stem cell division. Cell.

[23] Pinchi E., Frati P., Aromatario M., Cipolloni L., Fabbri M., La Russa R., Maiese A., Neri M., Santurro A., Scopetti M. (2019). miR-1, miR-499 and miR-208 are sensitive markers to diagnose sudden death due to early acute myocardial infarction. J. Cell. Mol. Med..

[24] Eisenberg I., Eran A., Nishino I., Moggio M., Lamperti C., Amato A.A., Lidov H.G., Kang P.B., North K.N., Mitrani-Rosenbaum S. (2007). Distinctive patterns of microRNA expression in primary muscular disorders. Proc. Natl. Acad. Sci. USA.

[25] Pinchi E., Frati A., Cantatore S., D’Errico S., Russa R., Maiese A., Palmieri M., Pesce A., Viola R.V., Frati P. (2019). Acute spinal cord injury: A systematic review investigating miRNA families involved. Int. J. Mol. Sci..

[26] Hanson E.K., Lubenow H., Ballantyne J. (2009). Identification of forensically relevant body fluids using a panel of differentially expressed microRNAs. Anal. Biochem..

[27] Neri M., Fabbri M., D’Errico S., Di Paolo M., Frati P., Gaudio R.M., La Russa R., Maiese A., Marti M., Pinchi E. (2019). Regulation of miRNAs as new tool for cutaneous vitality lesions demonstration in ligature marks in deaths by hanging. Sci. Rep..

[28] Odriozola A., Riancho J.A., de la Vega R., Agudo G., García-Blanco A., de Cos E., Fernández F., Sañudo C., Zarrabeitia M.T. (2013). MiRNA analysis in vitreous humor to determine the time of death: A proof-of-concept pilot study. Int. J. Leg. Med..

[29] Liberati A., Altman D.G., Tetzlaff J., Mulrow C., Gøtzsche P.C., Ioannidis J.P., Clarke M., Devereaux P.J., Kleijnen J., Moher D. (2009). The PRISMA statement for reporting systematic reviews and meta-analyses of studies that evaluate healthcare interventions: Explanation and elaboration. BMJ.

[30] Wang H., Mao J., Lib Y.B., Luo H., Wu J., Liao M., Liang W., Zhang L. (2013). 5 miRNA expression analysis in postmortem interval (PMI) within 48h. Forensic Sci. Int. Genet. Suppl. Ser..

[31] Tu C., Du T., Ye X., Shao C., Xie J., Shen Y. (2019). Using miRNAs and circRNAs to estimate PMI in advanced stage. Leg. Med..

[32] Li W.C., Ma K.J., Zhang P., Wang H.J., Shen Y.W., Zhou Y.Q., Zhao Z.Q., Ma D., Chen L. (2010). Estimation of postmortem interval using microRNA and 18S rRNA degradation in rat cardiac muscle. Fa Yi Xue Za Zhi.

[33] Pan H., Zhang H., Lü Y.H., Ma J.L., Ma K.J., Chen L. (2014). Correlation between five RNA markers of rat’s skin and PMI at different temperatures. Fa Yi Xue Za Zhi.

[34] Lv Y.H., Ma K.J., Zhang H., He M., Zhang P., Shen Y.W., Jiang N., Ma D., Chen L. (2014). A time course study demonstrating mRNA, microRNA, 18S rRNA, and U6 snRNA changes to estimate PMI in deceased rat’s spleen. J. Forensic Sci..

[35] Ma J., Pan H., Zeng Y., Lv Y., Zhang H., Xue A., Jiang J., Ma K., Chen L. (2015). Exploration of the R code-based mathematical model for PMI estimation using profiling of RNA degradation in rat brain tissue at different temperatures. Forensic Sci. Med. Pathol..

[36] Lü Y.H., Li Z.H., Tuo Y., Liu L., Li K., Bian J., Ma J.L., Chen L. (2016). Correlation between RNA Degradation Patterns of Rat’s Brain and Early PMI at Different Temperatures. Fa Yi Xue Za Zhi.

[37] Lv Y.H., Ma J.L., Pan H., Zhang H., Li W.C., Xue A.M., Wang H.J., Ma K.J., Chen L. (2016). RNA degradation as described by a mathematical model for postmortem interval determination. J. Forensic Leg. Med..

[38] Lv Y.H., Ma J.L., Pan H., Zeng Y., Tao L., Zhang H., Li W.C., Ma K.J., Chen L. (2017). Estimation of the human postmortem interval using an established rat mathematical model and multi-RNA markers. Forensic Sci. Med. Pathol..

[39] Tu C., Du T., Shao C., Liu Z., Li L., Shen Y. (2018). Evaluating the potential of housekeeping genes, rRNAs, snRNAs, microRNAs and circRNAs as reference genes for the estimation of PMI. Forensic Sci. Med. Pathol..

[40] Kuai J.-X., Liu Y., Zhang Y.W. (2008). A study on the relationship between the degradation of tubulin in cardiac muscle and lung of rat and the postmortem interval. Chin. J. Forensic Med..

[41] Sharma S., Singh D., Kaul D. (2015). AATF RNome has the potential to define post mortem interval. Forensic Sci. Int..

[42] Risoluti R., Canepari S., Frati P., Fineschi V., Materazzi S. (2019). “2 (n) Analytical Platform” to Update Procedures in Thanatochemistry: Estimation of Post Mortem Interval in Vitreous Humor. Anal. Chem..

[43] Henssge C., Madea B. (2007). Estimation of the time since death. Forensic Sci. Int..

[44] Giles S.B., Harrison K., Errickson D., Márquez-Grant N. (2020). The effect of seasonality on the application of accumulated degree-days to estimate the early post-mortem interval. Forensic Sci. Int..

[45] Payne J.A. (1965). A Summer Carrion Study of the Baby Pig Sus Scrofa Linnaeus. Ecology.

[46] Gill-King H., Haglund W.D., Sorg M.H. (1997). Chemical and ultrastructural aspects of decomposition. Forensic Taphonomy: The Postmortem Fate of Human Remains.

[47] Mann R.W., Bass W.M., Meadows L. (1990). Time since death and decomposition of the human body: Variables and observations in case and experimental field studies. J. Forensic Sci..

[48] Mathur A., Agrawal Y.K. (2011). An overview of methods used for estimation of time since death. Aust. J. Forensic Sci..

[49] Wilk L.S., Hoveling R.J.M., Edelman G.J., Hardy H.J.J., van Schouwen S., van Venrooij H., Aalders M.C.G. (2020). Reconstructing the time since death using noninvasive thermometry and numerical analysis. Sci. Adv..

[50] Fais P., Mazzotti M.C., Teti G., Boscolo-Berto R., Pelotti S., Falconi M. (2018). HIF1α protein and mRNA expression as a new marker for post mortem interval estimation in human gingival tissue. J. Anat..

[51] Tao L., Ma J., Han L., Xu H., Zeng Y., Yehui L., Li W., Ma K., Xiao B., Chen L. (2018). Early postmortem interval estimation based on Cdc25b mRNA in rat cardiac tissue. Leg. Med..

[52] Elghamry H.A., Mohamed M.I., Hassan F.M., Abdelfattah D.S., Abdelaal A.G. (2017). Potential use of GAPDH m-RNA in estimating PMI in brain tissue of albino rats at different environmental conditions. Egypt. J. Forensic Sci..

[53] Kim J.Y., Kim Y., Cha H.K., Lim H.Y., Kim H., Chung S., Hwang J.J., Park S.H., Son G.H. (2017). Cell death-associated ribosomal RNA cleavage in postmortem tissues and its forensic applications. Mol. Cells.

[54] Madea B. (2005). Is there recent progress in the estimation of the postmortem interval by means of thanatochemistry?. Forensic Sci. Int..

[55] Lijiang L., Xiji S., Liang R., Hongyan Z., Yan L., Wei L., Cheng Z., Liang L. (2007). Determination of the early time of death by computerized image analysis of DNA degradation: Which is the best quantitative indicator of DNA degradation?. J. Huazhong Univ. Sci. Technol..

[56] Li W.C., Ma K.J., Lv Y.H., Zhang P., Pan H., Zhang H., Wang H.J., Ma D., Chen L. (2014). Postmortem interval determination using 18S-rRNA and microRNA. Sci. Justice.

[57] Poór V.S., Lukács D., Nagy T., Rácz E., Sipos K. (2016). The rate of RNA degradation in human dental pulp reveals postmortem interval. Int. J. Leg. Med..

[58] Sampaio-Silva F., Magalhães T., Carvalho F., Dinis-Oliveira R.J., Silvestre R. (2013). Profiling of RNA Degradation for Estimation of Post Morterm Interval. PLoS ONE.

[59] Nolan T., Hands R.E., Bustin S.A. (2006). Quantification of mRNA using real-time RT-PCR. Nat. Protoc..

[60] Zhang H., Zhang P., Ma K.J., Lv Y.H., Li W.C., Luo C.L., Li L.L., Shen Y.W., He M., Jiang J.Q. (2013). The selection of endogenous genes in human postmortem tissues. Sci. Justice.

[61] Koppelkamm A., Vennemann B., Fracasso T., Lutz-Bonengel S., Schmidt U., Heinrich M. (2010). Validation of adequate endogenous reference genes for the normalisation of qPCR gene expression data in human post mortem tissue. Int. J. Leg. Med..

[62] Bauer M., Polzin S., Patzelt D. (2003). Quantification of RNA degradation by semi-quantitative duplex and competitive RT-PCR: A possible indicator of the age of bloodstains?. Forensic Sci. Int..

[63] Li C., Wang Q., Zhang Y., Lin H., Zhang J., Huang P., Wang Z. (2016). Research progress in the estimation of the postmortem interval by Chinese forensic scholars. Forensic Sci. Res..

[64] Heinrich M., Matt K., Lutz-Bonengel S., Schmidt U. (2007). Successful RNA extraction from various human postmortem tissues. Int. J. Leg. Med..

[65] Preece P., Cairns N. (2003). Quantifying mRNA in postmortem human brain: Influence of gender, age at death, postmortem interval, brain pH, agonal state and inter-lobe mRNA variance. Brain Res. Mol. Brain Res..

[66] Young S.T., Wells J.D., Hobbs G.R., Bishop C.P. (2013). Estimating postmortem interval using RNA degradation and morphological changes in tooth pulp. Forensic Sci. Int..

[67] Heinrich M., Lutz-Bonengel S., Matt K., Schmidt U. (2007). Real-time PCR detection of five different ‘endogenous control gene’ transcripts in forensic autopsy material. Forensic Sci. Int. Genet..

[68] Scrivano S., Sanavio M., Tozzo P., Caenazzo L. (2019). Analysis of RNA in the estimation of postmortem interval: A review of current evidence. Int. J. Leg. Med..

[69] Lü Y.H., Ma K.J., Li Z.H., Gu J., Bao J.Y., Yang Z.F., Gao J., Zeng Y., Tao L., Chen L. (2016). Correlation between RNA Expression Level and Early PMI in Human Brain Tissue. Fa Yi Xue Za Zhi.

[70] Tozzo P., Scrivano S., Sanavio M., Caenazzo L. (2020). The Role of DNA degradation in the estimation of post-mortem interval: A systematic review of the current literature. Int. J. Mol. Sci..

[71] Fineschi V., Picchi M.P., Tassini M., Valensin G., Vivi A. (1990). 1H-NMR studies of postmortem biochemical changes in rat skeletal muscle. Forensic Sci. Int..

[72] Zubakov D., Boersma A.W., Choi Y., van Kuijk P.F., Wiemer E.A., Kayser M. (2010). MicroRNA markers for forensic body fluid identification obtained from microarray screening and quantitative RT-PCR confirmation. Int. J. Leg. Med..

[73] Di Nunno N.R., Costantinides F., Bernasconi P., Bottin C., Melato M. (1998). Is flow cytometric evaluation of DNA degradation a reliable method to investigate the early postmortem period?. Am. J. Forensic Med. Pathol..

[74] Johnson L.A., Ferris J.A.J. (2002). Analysis of postmortem DNA degradation by single-cell gel electrophoresis. Forensic Sci. Int..

[75] Chen J.H., Inamori-Kawamoto O., Michiue T., Ikeda S., Ishikawa T., Maeda H. (2015). Cardiac biomarkers in blood, and pericardial and cerebrospinal fluids of forensic autopsy cases: A reassessment with special regard to postmortem interval. Leg. Med..

[76] Kikuchi K., Kawahara K.I., Biswas K.K., Ito T., Tancharoen S., Shiomi N., Koda Y., Matsuda F., Morimoto Y., Oyama Y. (2010). HMGB1: A new marker for estimation of the postmortem interval. Exp. Ther. Med..

[77] Poloz Y.O., O’Day D.H. (2009). Determining time of death: Temperature-dependent postmortem changes in calcineurin A, MARCKS, CaMKII, and protein phosphatase 2A in mouse. Int. J. Leg. Med..

[78] Kumar S., Ali W., Singh U.S., Kumar A., Verma A., Bhattacharya S. (2014). The effect of elapsed time on the cardiac Troponin-T (cTnT) proteolysis in case of death due to burn: A study to evaluate the potential forensic use of cTnT to determine the postmortem interval. Sci. Justice.

[79] Kimura A., Ishida Y., Hayashi T., Nosaka M., Kondo T. (2011). Estimating time of death based on the biological clock. Int. J. Leg. Med..

